# 欧洲呼吸学会和欧洲胸外科医师学会恶性胸膜间皮瘤诊疗指南

**DOI:** 10.3779/j.issn.1009-3419.2010.10.14

**Published:** 2010-10-20

**Authors:** A. Scherpereel, P. Astoul, P. Baas, T. Berghmans, H. Clayson, P. de Vuyst, H. Dienemann, F. Galateau-Salle, C. Hennequin, G. Hillerdal, C. Le Pe'choux, L. Mutti, J-C. Pairon, R. Stahel, P. van Houtte, J. van Meerbeeck, D. Waller, W. Weder, 作庆 宋, 萧洪 徐

**Affiliations:** 1 For a full list of affiliations, please see the Acknowledgements section; 2 天津医科大学总医院，天津市肺癌研究所，天津市肺癌转移与肿瘤微环境重点实验室

**Keywords:** 石棉, 肿瘤, 指南, 间皮瘤，胸膜, 治疗

## Abstract

恶性胸膜间皮瘤（malignant pleural mesothelioma, MPM）是一种罕见肿瘤，但发病率正逐渐上升，且预后较差。2008年，欧洲呼吸学会（European Respiratory Society, ERS）和欧洲胸外科医师学会（European Society of Thoracic Surgeons, ESTS）特别工作组召集各方专家计划制定MPM诊治经验及更新指南。

为了使MPM得到及时准确的诊断，专家推荐对患者实施胸腔镜检查，有手术禁忌和胸腔粘连的病例除外。约10%的病例采用标准染色方法无法获得满意的结果。因此我们推荐在胸膜活检的基础上，采用特异性免疫组化标志物。由于目前缺乏一个统一的、切实有效的分期系统，我们推荐应用最新的TNM分期，并且提出三个阶段的治疗前评估。在MPM的治疗中，患者的体力状态评分和组织亚型是目前唯一的、具有重要临床价值的预后因素。在临床试验中，应对其它潜在因素进行初步探讨并予以报道。MPM对化疗高度耐受，仅有少数患者可接受根治性手术。本文对新的治疗方法和策略进行了综述。

目前由于最佳综合治疗的资料有限，适合采用多种方案联合治疗策略的患者应被纳入专业机构的前瞻性试验中。

## 内容

**前言**………………………………………………………………………………………………………………………………………C24

**方法**………………………………………………………………………………………………………………………………………C24

**恶性胸膜间皮瘤（Malignant pleural mesothelioma, MPM）的流行病学**………………………………………C24

 MPM的相关危险因素有哪些………………………………………………………………………………………………………………C24

 MPM的流行病学的未来趋势是什么………………………………………………………………………………………………………C26

 石棉暴露的评估方法有哪些………………………………………………………………………………………………………………C26

 MPM筛查的依据是什么……………………………………………………………………………………………………………………C26

**MPM的诊断**…………………………………………………………………………………………………………………………………C27

 从临床角度考虑…………………………………………………………………………………………………………………………C27

 从病理学角度考虑………………………………………………………………………………………………………………………C27

**分期、治疗前检查和预后因素**………………………………C28

 采用哪种分期分类方法？………………………………………………………………………………………………………………C28

 最简单的治疗前分期检查方法是什么？………………………………………………………………………………………………C28

 重要的预后因素有哪些？………………………………………………………………………………………………………………C29

**MPM的治疗**………………………………………………………………………………………………………………………………C30

 MPM的手术治疗…………………………………………………………………………………………………………………………C30

 MPM的放疗………………………………………………………………………………………………………………………………C32

 MPM的化疗………………………………………………………………………………………………………………………………C33

 MPM的综合治疗…………………………………………………………………………………………………………………………C35

 MPM对症治疗……………………………………………………………………………………………………………………………C36

**致谢**………………………………………………………………………………………………………………………………………C37

**参考文献**…………………………………………………………………………………………………………………………………C38

## 前言

MPM是一种高侵袭性肿瘤，曾被认为非常罕见，近年来，MPM已经成为一个很重要的课题^[1]^。石棉暴露是参与MPM发病机理的主要因素，这可以解释20世纪60年代以来MPM发病率的增高。尽管2005年在欧洲以及多数其它发达国家已经禁止应用石棉，但是流行病学预测表明：在未来10年中，MPM的发病率将继续增加并达到顶峰^[1, 2]^。此外，目前一些国家仍然生产大量石棉，前五名国家包括：俄罗斯（到目前为止最大的生产国）、中国、哈萨克斯坦、巴西和加拿大。这些国家以及印度等其它发展中国家和欠发达的国家仍在应用石棉^[3]^。

MPM的诊断比较困难，因为从石棉暴露到发病，大概有30年-40年的潜伏期，并且通过胸膜活检，甚至应用免疫组化技术，MPM与胸膜良性疾病或腺癌胸膜转移之间的鉴别诊断也比较困难^[4, 5]^。因为MPM患者的预后较差，甚至源自SPLF（the French speaking Society for Chest Medicine）、BTS（the British Thoracic Society）、ESMO（the European Society of Medical Oncology）的最新指南尚未明确MPM最佳治疗方案，因此提示MPM在较长的时间内仍将是一个主要的公众健康难题^[4-7]^。

因此，欧洲呼吸学会（European Respiratory Society, ERS）和欧洲胸外科医师学会（European Society of horacic Surgeons, ESTS）合作，从2007年5月至2008年11月，召集来自不同科研机构的间皮瘤领域的专家制定MPM的诊疗建议，以期为临床医师提供简洁、准确、最新的MPM诊疗指南。

## 方法

专家们依据如下数据库对1990年-2009年的文献进行了系统分析：Medline（国家医学图书馆，美国）、Embase（艾斯维尔，荷兰）、循证医学图书馆（英国）、国家指南库（美国）、HTA数据库（国际网络卫生技术评估）、美国国立卫生研究所、国际胸膜间皮瘤项目（世界卫生组织数据库）。采用以下关键词作为文献的检索条目：pleura、cancer、mesothelioma、guidelines、asbestos、treatment、surgery、chemotherapy和radiotherapy。然而，化疗的文献检索期限为1965年-2009年。

专家根据美国胸科医师学会（American College of Chest Physicians, ACCP）提供的基于循证医学的官方提议对各项建议进行分级^[8]^（补充数据中的表 1）。简而言之，ACCP各项建议的强度取决于以下因素：1）获益、风险及负担间的权衡（明确，为1类；不明确，为2类）；2）疗效相关的证据的质量，分级如下：呈现一致疗效的随机对照研究（randomised controlled trials, RCT）；或者有很明显的治疗效果的观察性研究；有局限性的RCT，或具有特殊疗效的观察性研究；或无特殊疗效的观察性研究和病例系列研究。因此，ACCP系统（补充数据中的表 1）产生了从很高级别（明确的获益/风险比，高质量的证据；1A级）到很低级别（不明确的获益/风险比，低质量的证据；2C级）的推荐。每项推荐均经所有专家投票表决：如果对某一建议少于85%的专家同意，该推荐将在重新讨论后予以修正。这些推荐详见随后的指南。

**1:** 免疫组化鉴别上皮间皮瘤和腺癌 Immunohistochemistry to separate epithelioid mesothelioma from adenocarcinoma

抗体	价值	间皮瘤	阳性率	腺癌	阳性
间皮瘤					
钙结合蛋白	必不可少	阳性（细胞核和胞质）	80%-100%	常阴性	5%-10%肺腺癌细胞质阳性
角蛋白CK5/6	有价值	阳性（胞质）	60%-100%	常阴性	2%-10%局灶阳性
WT-1	有价值	阳性（细胞核）	43%-93%	肺腺癌阴性	0%
EMA	有价值	阳性（细胞膜）	60%-100%	阳性（细胞质）	70%-100%
Podoplanin	有价值	阳性（细胞膜）	80%-100%	常阴性	7%局灶性阳性
肺腺癌					
CEA单抗	很有价值	几乎全部阴性	0%	阳性（细胞质）	50%-90%
CD15	有价值	不表达	0%	阳性（细胞膜）	50%-70%局灶性阳性
Ber-EP4	很有价值	阳性/阴性（细胞膜）	局灶性阳性率为20%	阳性（细胞膜）	95%-100%
TTF-1	很有价值	不表达	0%	阳性（细胞核）	70%-85%肺腺癌
B72.3	很有价值	几乎无阳性表达	＜1%	阳性（细胞质）	70%-85%肺腺癌
乳腺癌					
ER	很有价值	不表达	0%	核染色阳性	≦70%
CK5/6: cytokeratin 5/6; WT-1: Wilms tumour antigen-1; EMA: epithelial membrane antigen; CEA: carcinoembryonic antigen; TTF-1: thyroid transcription factor-1; ER: endoplasmic reticulum marker. Note: Reprinted with permission from the copyright holder ©ERS Journals Ltd 2010 CK5/6：钙结合蛋白5/6；WT-1:肾母细胞瘤抗原-1；EMA:上皮细胞膜抗原; CEA:癌胚抗原; TTF-1:甲状腺转录因子-1; ER:内质网标志物。 注：本表得到版权所有者©ERS Journals Ltd 2010复制许可					

值得注意的是，ACCP的作者们在提出这些指南时强调：“无论这些建议为哪一级别，临床医师须考虑当地患者和单个患者的情况，以及患者的价值观，应依靠自己的判断来制定个体化方案。然而，一般来说，随着建议从2C级到1A级，临床医师需要仔细斟酌专家的建议^[8]^。”这可以解释ERS/ESTS的专家为什么在其推荐指南中应用了不同的术语（如“应该”或者“可能”），以便读者在临床实践中调整各推荐的强度。

## 恶性胸膜间皮瘤的流行病学

### MPM相关的危险因素有哪些？

**石棉**

石棉是MPM的首要致病因素。这主要包括6种可形成极细纤维的硅酸盐矿物：温石棉、青石棉、铁石棉、直闪石石棉、透闪石棉石和阳起石石棉。温石棉属于蛇纹石类矿物，其它属于角闪石矿物。在肺内，温石棉的生物持续性较角闪石短。温石棉、铁石棉和青石棉已被广泛用于工业用途。

第一篇阐述石棉和MPM关系的文章发表于20世纪60年代^[9]^。由于大部分石棉暴露与工作相关，所以间皮瘤在大部分病例中为一种职业病，其背景发病率较低。由于过去石棉暴露多见于以男性劳动力为主的职业，所以目前MPM的发病率在男性高于女性。例如，根据法国国家间皮瘤监测项目，职业性石棉暴露所致的危险分数在男性>80%，在女性＜40%^[10]^。职业性石棉暴露所致的危险分数的性别差异在其它国家亦有报道。

在过去的十年中，人们观察到间皮瘤病例的接触史发生了改变，从最初的石棉工人（处理生石棉材料）到经常接触石棉产品或处理石棉的一线工人，如建筑工人、电工、水管工和暖气工人。尽管第一组人群发生间皮瘤的风险最高，但目前后一组人群中罹患MPM的人数更多。

环境性间皮瘤与某些地区的自然暴露相关，如在一些地区，石棉（通常为透闪石）以土壤中的地质成分存在（土耳其、科西嘉岛、塞浦路斯和新喀里多尼亚），在有些地区，人们用石棉粉刷房屋墙壁，或者与靠近石棉矿或石棉工厂生活的人们的临近暴露相关^[11, 12]^。准职业性病例是指石棉工人的家属，主要是由于工作服引起的家庭暴露。

石棉暴露与MPM之间的剂量-效应关系已得到证实，但不可能确定累积石棉暴露量的阈值，低于这个阈值即不会增加罹患MPM的风险^[13]^。因此，所有暴露于石棉的个体均为危险人群。石棉暴露后，MPM的平均（范围）潜伏期约为40年（15年-67年）。在对1 690例患者的回顾性分析中，潜伏期＞15年者占所有病例的99%^[14]^。

在商用石棉纤维中，青石棉、铁石棉比白石棉具有更高的致胸膜癌的能力。目前尚不能排除短石棉纤维的致癌潜能。

MPM可见于不合并其它石棉相关疾病（肺或胸膜纤维化）的暴露个体中。在大多数病例中，胸膜斑是既往石棉暴露的一个征象，有报告称其与间皮瘤的高危险性相关。的确，与普通人群相比，间皮瘤更常见于伴有胸膜斑的个体，因为这两种疾病与石棉暴露密切相关。有一些尸检报告或队列研究已经报道了这种相关性。但也有研究未发现该相关性。在澳大利亚的青石棉采矿和加工小镇威特努姆的一项癌症预防项目表明，通过在项目初始校正首次暴露时间、累计暴露量和年龄发现，胸膜增厚与胸膜间皮瘤的风险增加并不相关^[15]^。该作者还报道称，腹膜间皮瘤在这一人群中有所增加^[15]^。因此，总体来说尚无明确的证据表明，仅胸膜斑即可增加胸膜间皮瘤的危险性。

证据

在全球MPM的人群中内，＞80%男性患者暴露于石棉，而女性患者暴露于石棉的比例却远远小于这一数字。MPM和石棉间有明确的剂量效应关系，但是该疾病亦可见于石棉累计暴露量较低的患者中。MPM主要见于职业性石棉暴露，但亦见于准职业性石棉暴露和环境石棉暴露。大多数闪石纤维，特别是青石棉以及铁石棉和透闪石，比白石棉纤维具有更高的致胸膜癌潜能。大多数工人为不同石棉类型的混合暴露。间皮瘤与白石棉暴露相关，但白石棉常存在角石棉纤维的混合和污染。目前尚不能排除短纤维石棉的致癌潜能。在大多数病例中，胸膜斑是既往石棉接触的一种征象。尚无明确的证据表明仅胸膜斑即可增加MPM的风险。MPM可见于未伴有任何其它石棉相关疾病的暴露患者中。

声明

石棉引起的MPM在女性患者中比例较低，其原因目前尚未阐明，有待进一步的研究，包括隐匿性石棉暴露的调查和/或病因的分析（2B级）

**其它因素**

除石棉外，MPM的其它潜在致病因素或协同因素包括：暴露于其它天然纤维（毛沸石和氟浅闪石）或人造纤维（耐火陶瓷）、电离辐射和猿猴空泡病毒40（SV40）。众所周知，烟草在间皮瘤的发生无关，已发表的数据表明，人造（vitreous）纤维，如矿物纤维（岩棉、玻璃棉和渣棉）对人类无致胸膜瘤的能力。遗传因素可增加MPM的易感性，可能有助于MPM的发生，与家族性间皮瘤有关。一项研究表明，在土耳其的Karain，遗传易感性可影响矿物纤维的致癌力，在那里毛沸石与MPM的高发病率密切相关^[16, 17]^。

证据

对于一些高水平的证据支持为MPM的致病因素有：毛沸石和治疗性放射（如对乳腺癌和淋巴瘤）。对于其它的因素或情况，仍然有一些争论或低水平的证据：耐火陶瓷纤维和SV40病毒。已发表的数据表明，矿物纤维（石棉、玻璃棉和渣棉）对人类无致胸膜癌的潜能。烟草对胸膜无致癌能力。

### MPM的流行病学的未来趋势是什么？

在世界不同国家中，MPM的发病率有显著差异^[18]^，从每年百万分之七（日本）到百万分之四十（澳大利亚）不等^[19]^，该病在欧洲的发病率不到百万分之二十。这种差异是合理的，主要是由于历史上石棉进口和消耗不同，而且诊断技术和对疾病的认识的不同亦可以导致差异。

流行病学家认为MPM的最高发病率会在未来十年内出现。20世纪90年代的初步预测最近被重新评价，最高发病率和病例数比原先预计的少^[2, 20-22]^。该病在欧洲的最高发病率预计会在2015年-2020年出现^[19]^，而有些国家可能已达发病高峰（美国和瑞典）。

证据

MPM的发病率在不同的国家有较大的区别，这也恰恰反映了在过去的几十年里不同国家石棉消费量的差异。因为MPM的潜伏期很长，不同国家减少或禁止石棉应用的时间也不同，故MPM的最高发病率的出现时间很难精确估计，而且在各国间也不尽相同。流行病学预测在未来的10年内欧洲的MPM发病率会继续增加。21世纪仍然继续应用石棉制品的国家，其MPM的发病率在近几十年内会增长。

### 石棉暴露的评估方法有哪些？

对累计石棉暴露的评估有几种不同的方法和工具，如职业问卷调查和工作/暴露的累积风险。由于该疾病的潜伏期长，以及缺乏空气中纤维水平的准确数据，精确的评估可能有困难，尤其是对于普通民众而不是有经验的职业环境卫生师或职业医师。

通过光镜或者电镜对生物标本[支气管肺泡灌洗液（bronchoalveolar lavage, BAL）和肺组织]进行矿物分析（mineral analyses, MA）可以提供残余石棉量的一些信息，尤其是对闪石这样的比白石棉对肺具有更长生物持续作用的石棉。由于MPM具有很长的潜伏期而且MPM与低剂量石棉暴露有关，所以MA不会总显示高水平的石棉纤维或小体。然而，当出现高剂量的纤维时常提示曾有未知的或者难以评估的石棉暴露史（如间接暴露）。MA亦可识别特殊的环境纤维（如阳起石）^[23]^。

大部分MPM病例与既往的职业暴露相关，在大部分的国家工人赔偿计划中，MPM被认为是一种职业病，由于MPM是一种严重的致命的疾病，所以社会保障对患者和家属具有重要意义。与其它职业性癌症相比，间皮瘤的报道较少。我们应该根据国家工人赔偿法案或者是其它相关的社会保障法案对MPM患者的既往暴露史进行系统地评估^[10]^。

证据

对MPM患者的石棉暴露的评估可采用不同的方法，主要是通过特殊的职业和环境问卷调查。

建议

石棉暴露的评估（主要通过特殊职业和环境问卷调查）是有意义的，且根据相关的全国性条例其可应用于社会保障和医学法学目的（1A级）。

声明

暴露的评估在特定的科学研究中亦具有重要意义。然而，其不具有治疗价值，如果没有职业卫生学家或职业医师的帮助，暴露的评估很难实施（专家的建议）。

以上原则同样需要对生物样本进行MA（支气管肺泡灌洗液或者是肺组织样本中石棉体或者石棉纤维的定量分析）。间皮瘤的临床治疗无需MA。

### MPM筛查的依据是什么？

在疾病的早期阶段通过筛查发现病变，则可以通过系统内外科治疗改善预后。迄今为止，根据现有MPM的资料（流行病学、预后和治疗）以及潜在筛查方法的性能（敏感性、特异性），尚不能明确大规模筛查的医学价值^[4, 5]^。

低剂量计算机断层（computed tomography, CT）扫描尚未被证实为MPM的早期筛查的有效工具：因为在1 045例石棉暴露者中没有任何一例胸膜间皮瘤是经过CT筛查出来的^[24]^。正电子发射断层扫描（positron emission tomography, PET）和核磁共振成像（magnetic resonance imaging, MRI）在恶性胸膜疾病的临床诊疗以及良、恶性胸膜疾病的鉴别诊断当中是有价值的，但不宜用于筛查。

一些生物标志物，如可溶性间皮素相关肽（soluble mesothelin related peptides, SMRP）和骨桥蛋白，目前还处于研究之中^[25-27]^。鉴于已有的生物学标记物的敏感性和特异性，以及该疾病的普遍流行，如果对所有石棉暴露个体进行MPM筛查，其假阳性率可能是真阳性率的数倍。因此，目前生物学标记物尚不能用作筛查工具^[4, 5]^。最近有研究评估了血清SMRP作为筛查工具的价值。在一项包括538例职业石棉暴露者的前瞻性研究中，其特异性较低，且假阳性率较高^[26]^。在这一队列研究中，没有发现一例间皮瘤，反而发现了一例肺癌和一例可疑贲门肿瘤的患者，尽管其中有15（大约3%）例伴有SMRP升高。这一事实意味着大量的患者需要用昂贵的可能有害的检查来随访多年^[27]^。尚无证据显示MPM的早发现对于患者的治愈甚至延长数月生存期有所帮助。作者认为，在SMRP的诊断的精确度以及其浓度水平与患者生存期长短和疾病死亡率之间的特定关系不确定时，SMRP不应该用于筛查^[26]^。

建议

目前尚无有价值的方法可以筛查MPM（1B级）。但对于特定的高危暴露人群，还是应该采用胸部影像学和（或）生物标志物作进一步评估（1B级）。

## MPM的诊断

### 从临床角度考虑

**有临床诊断标准吗？**

建议

MPM的临床表现通常是非特异性的和隐匿的，因此，即使对于既往石棉暴露的患者，仅临床表现也不能作为诊断标准（1A级）。

**有特定的影像学诊断标准吗？**

建议

胸部X光片通常显示一侧胸腔积液或胸膜增厚。但胸片单独不能用于诊断MPM（1A级）^[28]^。

胸部CT也不是诊断MPM的金标准，但弥漫性或结节性的胸膜增厚可以提示MPM（1A级）^[28, 29]^。

声明

MRI对于间皮瘤的诊断无益（1B级）^[29]^。PET扫描不能用于诊断间皮瘤^[29-31]^（1C级）。

**胸腔镜在MPM的诊断中的作用？**

当临床和影像学检查怀疑间皮瘤时，最好的确诊方法是行胸腔镜检查，因胸腔镜可以获得明确的病理学组织标本。

建议

除非有手术禁忌证或胸膜粘连，建议行胸腔镜检查以诊断MPM（1A级）。

### 从病理学角度考虑

间皮瘤是指一种排列于浆膜腔的间皮细胞起源的恶性肿瘤，其准确诊断有赖于组织病理学检查。然而诊断并不容易，因为间皮瘤是一种有着多种组织病理学特点的异质肿瘤，而这些特点常导致误诊。此外，胸膜也是转移性疾病的常见部位。

宏观上，间皮瘤在其自然的生长过程中是不断变化的，这取决于它何时被发现。随着胸膜间皮瘤的进展，它们的大体外观也在某种程度上更提示为MPM，尽管其它恶性肿瘤也表现为间皮瘤的外观（胸腺瘤、癌、淋巴瘤、血管肉瘤等）。MPM的镜下特点在新的胸膜肿瘤的国际分类中有很明确的定义^[32]^。然而，大部分间皮瘤具有不同且易导致误诊的外观，类似于良性胸膜病变或转移性病变，而后者在人群中比间皮瘤更常见。因此，最常见的胸膜转移肿瘤来源于肺癌或乳腺癌（分别为7%-15%和7%-11%），而在标准HE染色切片上后者的外观常易与间皮瘤混淆。诊断难题也常见于胸膜良性炎症或者是反应性病变，而后者的发病年龄与MPM的发病年龄可能一致（心衰、胶原病、肺炎、消化疾病例如肝硬化等所导致的胸腔积液）。这些病变通常是继发性的，可导致间皮细胞的不典型增生，也常易与间皮瘤混淆。在间皮瘤调查的国家项目（1998-2007）中，法国病理学会进行了间皮瘤辅助诊断的验证试验，最初所诊断的病例的误诊占13%^[10]^。

**哪个样本对应哪种临床表现？**

MPM的首发临床体征常为胸腔积液，故细胞学检查常为首选诊断检查。

建议

由于细胞学诊断具有较高的误诊率，因此不推荐单独应用细胞学诊断作为间皮瘤的诊断依据（1B级）。

推荐先行间皮瘤的细胞学检查，再行组织学检查（1B级）。

间皮瘤的复发和转移可以单独采用细胞学确定，这一建议与国际间皮瘤协会的提议一致（1B级）。

间皮瘤的诊断，无论是细针穿刺活检（Abrams或Castelain细针）还是细胞学诊断面临同样的问题。只有当病理是来自于具有代表性的肿瘤组织，而且量足以进行免疫组织学特征检查，有恰当的临床、放射学和/或外科方面的资料时，我们才能做最终诊断。

建议

胸腔镜是首选的确诊检查，该检查有助于我们全面检查胸膜，获取较充足的活检组织（足够的脂肪和/或肌肉组织以确定是否有肿瘤侵润），其诊断率＞90%（1A级）。

由于细针穿刺活检敏感性不高（＜30%），故不推荐其作为间皮瘤的首选诊断方法（1A级）。

建议胸膜活检时获取正常的和病变的胸膜组织（1C级。不推荐单独应用冰冻切片诊断MPM（1B级）。

**采用哪种分期分类法？**

**建议**

推荐采用WHO2004分类方法作为间皮瘤分类方法（1A级）^[32]^。这一分类标准为诊断、预后和治疗提供依据。国际间皮瘤协会更新的分类标准将在2009年出台。

**是否应该在形态学检查的基础上增加免疫组化检查？哪种组织病理类型应选用哪种免疫组化标记物及使用多少种抗体？**

建议

推荐MPM的诊断常基于免疫组化检查结果（1A级）。

国际间皮瘤协会已经提出了多种建议。免疫组化检查结果取决于间皮瘤的肿瘤亚型是上皮样的还是肉瘤样的。

建议

为了从腺癌中分辨出上皮样间皮瘤，指南推荐采用两种具有间皮瘤阳性诊断价值的标志物[核标志物，如抗钙网膜蛋白和抗Wilms瘤抗原1抗体，或者膜标志物抗上皮膜抗体（EMA），对于上皮样间皮瘤，可采用抗细胞角蛋白抗体（CK）5/6，抗D2-40（podoplain）或抗间皮素抗体等]，以及两种具有阴性诊断价值的标志物（抗Ber-EP4抗体，一种膜标志物；抗甲状腺转录因子1抗体，一种核标志物；或抗癌胚抗原单克隆抗体、抗B72-3抗体、抗MOC-31抗体、抗雌激素/孕酮抗体、抗EMA抗体、胞浆染色）来协助诊断（1A级）。在不同来源的抗体中，需采用敏感性最小值在60%-70%的抗体。不推荐采用抗CK-70和抗CK-20抗体来诊断间皮瘤（1A级）。抗体需满足的条件见表 1。

为了鉴别肉瘤样间皮瘤与鳞癌和移行细胞癌（表 2），推荐采用两种广谱的抗角蛋白抗体和两种具有阴性预测价值的标志物（如抗CD34抗体和抗B细胞淋巴瘤2抗体标志物、抗结蛋白抗体、抗S100抗体）以明确诊断（1A级）。单一抗体的免疫染色阴性并不能排除间皮瘤诊断（1C级）。

**2:** 免疫组化鉴别肉瘤样间皮瘤与鳞癌、移行细胞癌 Immunohistochemistry for separating sarcomatoid mesothelioma from squamous and transitional cell carcinoma

抗体	价值	间皮瘤	阳性率	鳞癌和移行细胞癌	阳性率
间皮瘤					
钙结合蛋白	有价值	阳性（细胞核和胞质强阳性）	80%-100%	常细胞质阳性	5%-40%
角蛋白CK5/6	无价值	阳性（胞质）	60%-100%	细胞质阳性	100%
WT-1	很有价值	阳性（细胞核）	43%-93%	阴性	0%
鳞癌					
P63	很有价值	几乎全部阴性	0%	阳性（细胞核）	≦100%
Ber-EP4	有价值	阳性/阴性	＜20%阳性	阳性（细胞质）	80%-100%
MOC31	有价值	阳性/阴性（胞膜阳性）	2%-10%	阳性（细胞膜）	97%-100%
CK5/6: cytokeratin 5/6; WT-1: Wilms tumour antigen-1. Note: Reprinted with permission from the copyright holder ©ERS Journals Ltd 2010 CK5/6：钙结合蛋白5/6；WT-1:肾母细胞瘤抗原-1。 注：本表得到版权所有者©ERS Journals Ltd 2010复制许可					

对于不典型的间皮细胞增生，目前尚无可用的免疫组化标志物来鉴别所见细胞的良恶性。

**是否有必要进行电镜和分子生物学检查？**

建议

电镜检查和分子生物学检查不宜常规用于确诊间皮瘤（1A级）。

声明

尚未发现冰冻胸膜肿瘤组织检查具有诊断或治疗价值（1A级）。

**对于疑似MPM，是否应该采取专家的建议?**

建议

在确诊MPM时，应听取独立专家小组的建议，尤其对于临床试验中病例或疑似该病的所有病例（1B级）。

## 分期、治疗前检查和预后因素

### 应采用哪种分期分类方法？

分期用来描述肿瘤累及的解剖范围，对于MPM至少有5种分期方式，最新的一种分期方式是由国际间皮瘤研究组织提出，并获得国际抗癌联盟（International Union Against Cancer, UICC）的认可^[33]^。（见补充性数据表 2）。这种分期方法最大的缺陷是通过目前影像技术判断T和N的程度不够准确。所以国际专家小组对胸膜间皮瘤的分期没有达成共识，他们强烈呼吁在前瞻性研究和TNM分期以及现有外科病理分期系统的基础上，建立新的精确统一的分期系统。

**建议**

在缺乏统一、可靠、准确的分期的情况下，专家提倡使用最新的UICC提出的TNM分期（1C级）^[33]^。

### 最简单的治疗前分期检测是什么？

专家小组提出以下假设：1)最佳的治疗前评估方法应该是简单而应用广泛，连贯而有逻辑，无不必要的侵入性检查手段，为患者提供合适的治疗；2）在采取不同的治疗方式前，应该分别评估患者个体的功能的和心理的适应性（如心脏和肺/或肺功能）；3）在病历档案中，对每例患者的石棉暴露要记录和深入研究。

**证据**

治疗前评估分为三个阶段，这些阶段在某种程度上是重叠的^[34]^。患者是否完全经历这三个阶段，主要取决于病程的结果和采取积极或保守治疗的结果。

第一阶段是考虑所有患者临床表现或诊断（表 3）。第二阶段考虑哪类患者可以接受积极治疗（表 4）。第三阶段是患者选择综合治疗或积极局部治疗（表 5）。专家的建议是仅在少数胸膜间皮瘤患者才考虑进行最后一步，这些在缺乏证据是就会被反映，反映不同机构的实践。在检查中要考虑纵隔镜、胸部MRI、电视胸腔镜（video-assisted thoracoscopy, VATS）、支气管超声细针活检（enobronchial ultrasound-fine needle aspiration, E(B)US-FNA）、PET扫描和腹腔镜。由于缺乏对照试验，尚无有关这些检查手段作用的确凿证据。

**3:** 间皮瘤患者临床表现/诊断时需考虑的因素 Parameters to be considered in all patients at presentation/diagnosis

检查	包括	确诊检查
人口学	年龄、性别和石棉暴露	
病史	体力状态、并发症、有/无胸痛、呼吸困难、体重或BMI改变	符合
体格检查	有/无“肺固缩”、皮肤结节	符合
影像学检查	胸部X片，后前位/侧位	胸部X线、吸气/呼气、胸腔引流前后对照
血液学检查	血红蛋白、白细胞、血小板、基本生化检查	
BMI: body mass index; PA: postero-anterior. Note: Reprinted with permission from the copyright holder ©ERS Journals Ltd 2010 BMI:身体质量指数；PA:后前位。 注：本表得到版权所有者©ERS Journals Ltd 2010复制许可		

**4:** 可接受某些积极治疗的患者的筛选 Investigations performed in patients likely to receive some form of active treatment

项目	内容	确诊检查
原发肿瘤	组织学活检确定病理特征	
胸部及上腹部CT扫描	在胸腔积液引流后，至少包括两个肾脏平面的强化CT	
肺功能检查	肺活量、第一秒用力呼气量	
骨扫描	非常规，只有怀疑骨转移时	CT/MRI确定骨转移部位
颅脑CT/MRI	非常规，只有怀疑脑转移时	
CT: computed tomography; MRI: magnetic resonance imaging. Note: Reprinted with permission from the copyright holder ©ERS Journals Ltd 2010 CT：计算机断层扫描；MRI：核磁共振。 注：本表得到版权所有者©ERS Journals Ltd 2010复制许可		

**5:** 可接受手术或多种方式联合治疗的患者的筛选 Investigations to be considered in patients who are candidates for surgery or multimodal treatment

检查	内容	意义	确诊检查
肺功能检查	用力肺活量和第一秒用力呼气量、一氧化碳弥散量	类似于肺癌术前检查	对于行肺切除，行肺灌注扫描
原发肿瘤	为诊断获得足够的组织		
膈肌	CT或MRI检查		
肺外检查，以除外“偶然性”M1	PET-CT，腹腔镜检查	根据各医疗机构的实践	可疑病灶的肺外活检
纵膈侵犯，除外T4，	纵隔镜检查	根据各医疗机构的实践	
N2/3淋巴结侵犯	同侧/对侧VATS，胸部MRI，钆加强E(B)US-FNA	正在进行研究	
DL, CO: diffusing capacity of the lung for carbon monoxide; FVC: forced vital capacity; FEV1: forced expiratory volume in 1 s; CT: computed tomography; MRI: magnetic resonance imaging; FDG-PET: 18-Fluor-2-deoxy-glucose positron emission tomography; VATS: video-assisted thoracic surgery; E(B)US-FNA: enobronchial ultrasoundfine needle aspiration. Note: Reprinted with permission from the copyright holder ©ERS Journals Ltd 2010 DL, CO:肺一氧化碳弥散量；FVC：用力肺活量；FEV1：一秒钟用力呼气量；CT：计算机断层扫描；MRI：核磁共振成像；FDG-PET：（18）F-氟去氧葡萄糖-电子发射断层扫描；VATS：电视胸腔镜手术；E(B)US-FNA：支气管内超声引导针吸活检术。 注：本表得到版权所有者©ERS Journals Ltd 2010复制许可			

对于第二阶段或更高阶段的患者，专家们认为：1）间皮瘤的诊断应明确，最好通过对活检标本行免疫组化和亚型鉴定；2）完成治疗前评估的时间应该尽可能的短；3）在有创操作前应该有近期（＜1个月）的影像学资料。还应该进一步的研究不同腔内技术（纵膈镜、VATS和EUS-FNA）的优劣性和新技术（PET-CT、EBUS-FNA）的价值。

**建议**

三个阶段治疗前评估基于临床观察、高超的临床操作和个体化治疗的基础之上（1C级）。

### 重要的预后因素有哪些？

预后因素是治疗前患者的临床特点或肿瘤的生物特性，无论治疗与否，其可影响患者预后。

**证据**

一些预后因素已经通过了一系列大型的、多中心实验并已得到独立验证^[35]^。其中，美国检测、流行病学、最终结果（Surveillance, Epidemiology and End Results, SEER）项目评估为有关1 475例病理类型确诊为间皮瘤患者的一项具有里程碑意义的回顾性系列研究，结果发现，年龄、性别、肿瘤分期、治疗和居住地区是重要的预后因素^[36]^。一些因素如体力状态、分期和体重减轻是许多肿瘤共有的预后因素；年龄、性别并未在所有研究中得到认可。症状和生活质量日益成为预后因素。非上皮细胞亚型始终与不良预后相关。低血红蛋白、高乳糖脱氢酶（lactose dehydrogenase, LDH）、白细胞计数高、血小板计数高与不良预后相关。与预后不良相关的新血清标志（如中位雌酮和骨桥蛋白）目前正在研究中^[37-39]^。基于以上不同因素形成了3种预后评分系统，并进行了前瞻性验证：CALGB（Cancer and Leukaemia Group B）和两个EORTC（European Organization for Research and Treatment of Cancer）（表 6）^[40, 41]^。后者的获批基于一项在体能状态好的患者中实施大型随机化疗试验的有关预后因素的多因素分析的结果^[42, 43]^。

**6:** 恶性间皮瘤的预后评分系统 Prognostic scoring systems in malignant mesothelioma

	第一作者[引用]	病例数	指标	预后佳	预后差
			PS评分	好	差
			年龄	＜75 yrs	＞75 yrs
CALGB	HERNDON[40]	337	胸痛	无	有
			血小板计数	＜400×10^12^·L^-1^	≧400×10^12^·L^-1^
			乳酸脱氢酶	＜500 IU·L^-1^	≧500 IU·L^-1^
			PS评分	0	1–2
EORTC	CURRAN[41]	204	病理类型	上皮的	非上皮的
			性别	女性	男性
			诊断	诊断明确	可疑诊断
			白细胞计数	＜8.3×10^9^·L^-1^	≧8.310^9^·L^-1^
			分期	Ⅰ–Ⅱ	Ⅲ–Ⅳ
			病理类型	上皮的	非上皮的
			确诊时间	＜50天	≧50天
EORTC^#^	VAN MEERBEECK[42]	250	血小板计数	＜350×10^12^·L^-1^	≧350×10^12^·L^-1^
			血红蛋白差异^▲^	＜1	＞1
			胸痛	无	有
			食欲减轻	无	有
CALGB: Cancer and Leukaemia Group B; EORTC: European Organization for Research and Treatment of Cancer; LDH: lactate dehydrogenase; WBC: white blood cell. ^#^: performance status 0–1 was an inclusion criterion for this series; ^▲^: difference between actual value and 16 g·dL^-1^ and 14 g·dL^-1^ in males and females, respectively. Note: Reprinted with permission from the copyright holder ©ERS Journals Ltd 2010 CALGB：癌症和B组白血病；EORTC：欧洲癌症研究治疗组织；LDH：乳酸脱氢酶；WBC：白细胞；^#^：这一研究的纳入标准为体力状态为0分-1分；^▲^：实际数值间的差异，男性和女性分别为16 g·dL^-1^和14 g·dL^-1^。 注：本表得到版权所有者©ERS Journals Ltd 2010复制许可					

**建议**

目前，患者体力状态评分和组织病理亚型是唯一的具有临床意义的预后因素，其常用于恶性间皮瘤患者的治疗（2A级）。

预后评估的其它参数，如年龄、性别、分期、是否有特定症状和血液学因素，应在临床试验中进行记录和报告（2A级）。

## MPM的治疗

### MPM的手术治疗

**减瘤胸膜剥脱术/胸膜切除术可控制症状的证据是什么？**

减瘤胸膜剥脱术/胸膜切除术效果显著，但不能完全清楚肉眼所见的胸膜癌。手术目的是通过去除脏层肿瘤组织以解除压迫所致肺不张，通过去除壁层肿瘤组织可缓解限制性通气不足并减轻胸壁疼痛。手术过程可通过开胸手术或VATS来完成。

证据

只有少量的证据支持减瘤手术。目前缺少随机试验，但在英国一项由癌症研究所支持的试验正在进行中，该试验比较VATS减瘤术与VATS下化学药物胸膜粘连固定的疗效。有几篇回顾性研究为减瘤胸膜切除术提供低水平的证据^[44-47]^。开胸手术造成的损害降低了患者的获益^[48]^；然而有限的但逐渐增多的资料表明VATS能更好的控制症状和改善生存期^[46]^。

建议

胸膜切除术/胸膜剥脱术达不到治愈目的，但可缓解症状，特别是对于化学性胸膜固定术无效、且有肺不张综合征的患者（2C级）。应优先考虑VATS方法（1C级）。

**MPM根治性手术的证据是什么？**

根治性手术的定义是指从半侧胸廓去除所有肉眼可见的肿瘤。通过胸膜外肺切除术（extrapleural pneumonectomy, EPP）切除整个胸膜、肺、心包膜、膈膜，并进行系统淋巴结清扫，可达到此目的。

证据

有关间皮瘤的根治性手术的疗效证据有限。在切除间皮瘤的患者中，唯一长期生存的幸存者已行根治性手术（EPP），作为综合治疗方案的一部分。已有的前瞻性研究和回顾性资料研究显示根治术后患者中位生存期为20个月-24个月^[49-51]^。资历较好的医院的术后死亡率降至5%以下^[51]^，而复发率仍较高，约低于50%。

建议

EPP仅应用作为综合治疗方案的一部分用于专门的临床机构的临床试验。

### MPM的放射治疗

**姑息性放疗对缓解疼痛的作用是什么？**

建议

姑息性放疗的主要目的是缓解疼痛，对于因侵及胸壁而引起疼痛的患者，可考虑应用姑息性放疗（2C级）。

**放疗在预防引流通道附近的胸壁种植中的作用是什么？**

BOUTIN等^[52]^曾报道，在胸腔引流或者胸腔镜检查以后4周内，放疗连续3天，一次7Gy，以防止引流通道附近的皮下转移的发生。然而，最近公布的随机对照试验比较了1998年-2004年间引流管口给予及时分三个阶段放疗21Gy的患者与61例给予最佳支持治疗的患者的疗效，结果发现，在引流通道附近转移性复发方面无差异^[53, 54]^。O’ROURKE^[53]^等通过以前的研究认为MPM引流通道部位的放疗不能减少肿瘤种植的发生率，这和CHAPMAN^[55]^等的结论一致。放射治疗技术的缺陷可以解释这些结果的差异，显然这也是一个争论点。

建议

预防性放疗的价值争议较大，因此专家没有给出任何建议。

**术后放疗的作用是什么？**

术后放疗的资料有限，且均来自于回顾性研究。

建议

胸膜切除术或胸膜剥离术后不推荐进行放射治疗（1A级）。EPP后放疗只能作为综合治疗方案的一部分用于专门的临床机构的临床试验（1A级）。

由于缺乏Ⅲ期的随机对照研究，专家建议进行一项前瞻性对照试验以评估EPP后辅助放疗的效果和耐受性。（最小剂量50 Gy，每天1.8 Gy-2 Gy）（1C级）。欧洲一项多中心随机研究正在进行中，以解决这个问题（SAKK研究）。回顾性研究似乎显示出辐射剂量效应，应进一步进行适形放疗技术的研究。RUSCH等[50]的一项研究表明，EPP后一侧胸腔给予54 Gy的辅助放疗，局部复发率为13%，仅局部（local-only）复发率为4%。然而在BALDINI的研究^[56]^中，经过调强放疗后，局部复发率为50%，仅局部复发率为13%。周围的正常结构（特别是心脏和肝脏）的限制导致放疗无法完全覆盖所有可能复发区域，以及给药总剂量和放疗技术可以解释这些差异。

**MPP在行EPP后调强放射的作用是什么？**

在EPP后给予调强放疗的辅助治疗，初步结果似乎令人满意，因为调强放疗具有较好的局部控制而且可保护正常组织，如肝脏和心脏。然而，近来有研究报告调强放疗并发严重的肺炎，因此除了临床实验外，调强放疗不应该被推荐；13例患者中有6例发生了严重的肺炎^[57]^。

通过最近的回顾性研究来预测发生放射性肺炎的风险，以下的肺剂量测定值（V20、V5和平均肺剂量）应明确。V20[两个肺的面积减去计划靶区体积（planned target volume, PTV）]应该少于15%，平均肺剂量应该少于10 Gy。这些剂量限制，同样也可以用于适形放疗。所有靶器官（临床靶区和靶区体积）和所有重要器官（对侧肺、心脏体积、脊髓、食道、肝、左肾、右肾）的剂量体积直方图（dose-volume histograms, DVH）均应明确。

声明

放射治疗的作用还需要进一步深入研究。最近的研究报道了放射治疗技术在局部控制肿瘤和放射毒性方面的重要性。

推荐

建议只在专门的临床中心进行放射治疗（专家建议）。

### MPM的化疗

用来回答下列问题所使用的方法已被描述^[4, 58]^，这些建议是基于：1）安大略癌症护理的网上建议^[59]^；2）BERGHMANS等所发表的荟萃分析文献^[60]^，这些文献已更新到2003年^[61]^，完全分析完成需要到2009年1月；3）由SPLF发表的法国学者关于MPM化疗的建议^[4, 58]^。

**化疗的益处已经得到证明了吗？**

目前只有一项随机对照研究评价了在MPM中化疗和安慰剂的疗效。研究结果发表在2007年美国临床肿瘤学会和欧洲克罗恩结肠炎协会会议上^[62]^。除在长春瑞滨亚组中发现生存优势外，未观察到化疗组和安慰剂组之间有生存差异。必须指出的是，根据随机研究和系统评估结果^[60, 61]^，对化疗选择的比较不够充分。此外，由于患者数量有限该研究被提前终止。由VOGELZANG^[63]^等和VAN MEERBEECK^[42]^等进行的随机研究表明，如果我们认为顺铂单药化疗无效，联合化疗包括顺铂和抗叶酸制剂、培美曲塞或雷替曲塞可延长生存期。的确，顺铂联合培美曲塞组（12.1个月）或顺铂联合雷替曲塞组（11.4个月）的中位生存期比既往文献报告（7个月-9个月）有明显延长。这与顺铂的单药化疗（9.3个月和8.8个月）在统计学上有差异，间接的证明了化疗有益。然而，尚无研究报道顺铂单药和单纯姑息性治疗的疗效比较。

补充资料表 3总结了随机（6项试验）和非随机研究中的化疗相关结果。在第一行可见三项随机Ⅲ期实验^[42, 62, 63]^。实验表明，尽管顺铂单药不能作为标准治疗方案，但顺铂联合培美曲塞或者雷替曲塞在缓解率和生存期方面均优于顺铂单药。需要注意的是，补充叶酸和维生素B_12_可减轻培美曲塞的血液学毒性。其它以顺铂为基础的化疗方案的缓解率较高，Ⅱ期研究的荟萃分析^[60, 61]^显示联合化疗的缓解率为25%-30%，联合方案包括：顺铂联合依托泊苷、顺铂联合多柔比星、顺铂联合吉西他滨、顺铂联合干扰素、奥沙利铂联合雷替曲塞（或吉西他滨或长春瑞滨）。在进一步的随机试验中，顺铂联合培美曲塞或雷替曲塞可以作为参考。在临床试验中纳入身体状况较好的患者仍需伦理学认可。

尚无随机研究表明，一线化疗失败后，二线治疗可影响生存期或生活质量。从一线治疗的随机试验随访资料间接提取的数据表明^[64]^，与控制症状相比，顺铂联合培美曲塞的二线化疗可提高生存期。这些数据需要随机研究来证实。二线治疗的数据较少（6项Ⅱ期研究^[65-70]^），尚不足以提议详尽的化疗方案。推荐将身体状态好的患者纳入临床试验，需经伦理学认可。

Ⅲ期实验表明，顺铂联合培美曲塞或雷替曲塞等一线联合化疗比单独应用顺铂的疗效好，缓解率较高且生存期延长（1级）。然而，BTS研究表明，与最佳支持治疗相比，化疗（单独应用长春瑞滨或丝裂霉素C，长春碱联合顺铂）没有生存优势（2级）。其它可能组合的研究，如顺铂+吉西他滨或依托泊苷或阿霉素也应进行（与最佳支持治疗或顺铂/培美曲塞或雷替曲塞相比）（专家意见）。非铂类方案的作用仍然需要进一步的阐明（2级）。尚无随机研究表明一线化疗失败后二线化疗对生存期或生活质量有益（除JASSEM等^[71]^进行的一项有关无疾病进展生存的Ⅲ期研究）。

建议

每个患者应该至少给予最佳支持治疗（1A级）。当决定给患者实施化疗时，如果患者的状况较好（根据东部肿瘤协作组的PS评分＞60和卡氏评分＜3），应该给予以铂类为基础，联合培美曲塞或雷替曲塞的一线化疗方案（1B级）。此外，患者可以纳入一线或二线临床试验。

由于对化疗疗效缺乏足够的证据，进行化疗时应该与患者及他们的家属逐项说明，就像其它的非治疗性目的的治疗方式一样（专家建议）。

**化疗应该在什么时候开始，应该持续多久？**

目前的文献缺乏最佳开始化疗的时间的结论。1）两项随机Ⅲ期临床试验发现一般情况较好的患者的总生存期较长^[42, 63]^。2）肿瘤体积越小化疗疗效越好^[72-74]^。3）一项小样本的随机实验比较了经过至少4周治疗控制症状后立即开始化疗与患者症状进展后才开始化疗的疗效。立即化疗患者的症状恶化前的时间（25周 *vs* 11周）和生存期（平均14个月 *vs* 10个月，1年生存率66% *vs* 36%）均有所延长，但是与对照组比较无统计学意义（p=0.1）^[75]^。

目前没有资料能够明确回答化疗的最佳持续时间。VOGELZANG等研究^[63]^表明，53%的患者可接受顺铂+培美曲塞化疗6个周期（1个-12个周期，5%超过8个周期）。Van MEERBEECK等^[42]^研究表明，顺铂+雷替曲塞平均周期为5个周期（1个-10个周期）。对稳定期患者给予超过6个周期化疗的可能优势，我们尚无数据。同非小细胞肺癌相比，我们建议如果出现疾病进展或3级-4级药物毒性或累积剂量的毒性，应停止化疗；疾病稳定或化疗反应良好的患者在化疗6个周期后应停止化疗。包括生物疗法在内的实验治疗，按预先说明的试验协议必须终止。尚无采用化疗或免疫调节剂维持治疗的疗效数据。

建议

化疗不宜延迟，而且应在功能性临床症状开始前实施（1C级）。

当出现疾病进展、3级-4级毒性反应或者累积剂量的毒性（1A级），或者化疗反应良好以及疾病稳定的患者在化疗6个周期后（2C级），应停止化疗。

**作为二线治疗的细胞毒性药物有效吗？**

一些文献特别关注了二线化疗的疗效^[65-71, 76, 77]^。其它文献很难说明其疗效，因为它们评估的患者都接受了一线和二线治疗，这些研究并没有考虑这一评估。化疗方案包括多柔比星、多柔比星+环磷酰胺、奥沙利铂+雷替曲塞或ZD 0473（铂类似物）无效。单独应用培美曲塞^[70]^、卡铂联合培美曲塞^[70]^以及顺铂、伊立替康联合丝裂霉素C^[69]^显示出较好的反应率。然而，在一项Ⅲ期随机试验中培美曲塞与最佳支持治疗比较表明，培美曲塞在疾病缓解率和和疾病进展时间方面得到了改善，但没有显示出任何的生存优势^[77]^。由于长春瑞滨显示一线活性，可能是二线治疗理想的药物。最近的一项63例患者的小样本研究报道反应率为16%，中位生存期为9.6个月^[77]^。因此，在二线化疗中尚无药物被证实有优势，应建议将身体状态较好的患者纳入临床试验。

建议

患者在一线化疗药物治疗后，如果呈现持续性症状缓解和客观反应^[72-74]^，复发时可使用相同的化疗方案（2C级）。

在其它情况下，鼓励患者进入临床试验（2C级）。

**生物疗法在MPM中的作用是什么？**

调控免疫系统活性的药物或对肿瘤有“特殊”作用的药物（靶向治疗）的研究结果总结见补充资料表 3。

免疫调节剂

在恶性间皮瘤的生物治疗中，干扰素和白细胞介素（interleukins, ILs）是主要的试验性药物。目前各临床试验的剂量、给药方法（胸膜内、皮下、肌肉和静脉）、药物类型和疾病分期各不相同，故对这些研究结论需要进一步验证。目前，干扰素和IL-2的单药治疗无效，也不推荐在临床试验之外使用。

在少量的患者中给予分枝杆菌疫苗观察到有趣的初步结果。在允许应用这种治疗方式之前，还需要进一步的证实。尚未证实豹蛙酶有效。

靶向治疗药物

一些生物靶向药物在肺癌、结肠癌和乳腺癌中有效。其在恶性间皮瘤中的研究较少。目前已进行试验的药物包括以下几种。

①沙利度胺（抗血管生成药物）：在包含40例患者的Ⅰ/Ⅱ期临床试验表明，11例患者疾病稳定时间＞6个月，中位生存期为230天^[78]^。然而，这些结果不能作为沙利度胺是一个有效的药物的依据^[78]^。②贝伐珠单抗（直接作用于血管内皮生长因子的单克隆抗体）：一项Ⅱ期随机研究比较了顺铂+吉西他滨联合或不联合贝伐珠单抗的疗效，结果显示，联合贝伐珠单抗并未提高生存率（25% *vs* 22%）和生存期（中位生存期15.6个月 *vs* 14.7个月，p=0.91）^[79]^。③吉非替尼：在一项包含42例患者的Ⅱ期实验研究中，患者每天口服吉非替尼500 mg，只有2例呈现客观缓解。作者认为吉非替尼对恶性间皮瘤患者无效^[80]^。④伊马替尼：一项Ⅱ期试验研究^[81]^以及美国临床肿瘤学会会议上的两项研究证实其无效^[82, 83]^。⑤厄洛替尼：在一项包含33例患者的Ⅱ期临床研究中未观察到患者客观缓解^[84]^。

推荐

除非在临床试验中，生物调节制剂、靶向药物、疫苗不宜用于MPM的治疗。

**应用什么评估标准来评估化疗在MPM中的疗效？**

通过临床标准（症状控制和生活质量）、影像学标准（CT扫描或PET）、生存标准（疾病进展时间和总生存期）可进行疗效评价。胸腔镜的疗效评估尚无报道。

肿瘤缓解的影像学评估标准

评估标准在不同的研究中各不相同，且不常被报道。参照CT扫描时间是胸膜固定后、化疗开始前，其系统化并不是强制性的，导致了反应评估的偏倚。大多数情况下评估时机的选择也缺乏规定。

今天，我们认为采用标准的胸部X光来评估化疗效果（指诊断部分）不是一种有价值的方法。

根据标准有不同的方法来评估客观缓解，WHO（两个垂直方法）或RECIST（一维方法）。这两种标准都不能用来评估恶性间皮瘤，恶性间皮瘤的发展本质是胸膜的总的表面^[85]^。目前建议应用RECIST标准（测量垂直于胸壁短直径）以评估MPM的客观缓解^[85-87]^。

根据PET标准来评估肿瘤客观缓解

CT扫描难以鉴别肿瘤组织与化疗后瘢痕组织。PET能同时评估肿瘤大小和摄取量。PET和CT扫描相结合，在同样的位置对患者进行检查，使这两项技术有更好的相关性。这种新的显像模式的作用仍需进行验证。对于临床试验，PET评估恶性间皮瘤疗效缺乏标准，可以考虑采用欧洲癌症治疗研究组织提出的建议标准^[88]^（补充数据表 4）。

生存期

在治疗方案中，总生存期是用来评估化疗有效性的唯一有价值的标准。

生活质量

建议考虑采用生活质量和症状控制来评估预后不良疾病的临床获益（有效/耐受），而且治疗对该疾病的生存期的影响尚不明了或极其微小。目前没有特殊的评分系统可用于评估生活质量，只能采用适用于恶性间皮瘤患者的改良的肺癌症状量表（Lung Cancer Symptom Scale, LCSS）^[89]^。

建议：采用CT扫描来评估MPM的治疗效果和随访情况。对于已行胸膜固定术的患者，为了更好的评估治疗效果，在进行化疗之前应进行CT扫描（1B级）。

改良的RECIST是评估治疗效果的最佳方法（1B级）。

PET扫描和生物学标志物用以评估MPM的治疗效果正在研究之中。

### 综合治疗

以下是EPP的适应症：1.胸膜活检证明是非肉瘤样MPM；2.临床/病理分期是T1-3、N0-1、M0（值得注意的是，有些中心也包括N2的患者，尽管N2的患者总生存率较低）；3.无其它并存疾病，如心血管疾病，且心肺功能储备较好的患者宜行肺切除术；4.患者适宜接受新辅助化疗或辅助化疗；5.患者可以接受一侧胸廓的辅助放射治疗；6.计算患者的EORTC和CALGB评分（表 6）有助于判断其是否为EPP的适应症，但是根据评分来预测良好预后的价值需要前瞻性临床试验验证。

**综合治疗模式的理论依据是什么？**

以前文献表明，因为没有完全切除肿瘤，单纯的外科手术不能治愈恶性间皮瘤。胸膜内层特别是在心包膜和纵隔边缘的1 cm-2 cm不能被切除。故所有外科操作均为R1切除^[50]^。因此，这是综合治疗的理论基础（证据水平：强/低水平的证据）。

由于临近肺、肝脏，心脏以及脊髓和食管等重要脏器，故对一侧胸腔完全放疗受到了限制。对于这样大的范围，实施总剂量超过54 Gy的照射是很困难的，因为这要求很高的治疗技术，并需要通过外科医生和病理学医师所见来进行定向^[90, 91]^（证据水平：强/低水平的证据）。

**综合治疗适用于哪些患者？**

由于手术和综合治疗的程度，在接受任何多模式的综合治疗前，患者均须接受深入检查，2004年以前由于缺乏有效的化疗方案，大多数综合治疗集中在手术后放疗。对患者的检查包括如下项目：①体格检查：单侧胸廓萎缩是晚期疾病的一个信号。肋骨和腹部无肿瘤生长征象。②肺功能检查：肺切除术后的肺功能数值应满足正常生活需要。③要有充足心功能储备，无肺动脉高压和心律不齐。（证据水平：低/中等水平证据）。④放射线检查以排除通过膈扩散至胸腔、对侧扩散以及多淋巴结受累及（证据水平：低/中等水平证据）。⑤组织学检查：上皮型MPM的预后最好（证据水平：低/高等水平证据）。⑥性别：尚无有力的资料证实不同性别间的治疗效果不同^[92]^（证据水平：强/低等水平证据）。

**最佳联合治疗的方法是什么？**

目前有较多术后化疗的文献报道。手术方式随切除的范围而异（膈肌完全切除、心包切除、补片修补等）。近来术前或术中化疗逐渐被广泛应用，有两项关于铂类联合抗叶酸制剂（培美曲塞）的研究，其中一项已经作为海报介绍^[93]^，然而EORTC 08031研究正在分析中^[94]^。也有关于术前或术后化疗联合热疗的报道，然而这种方法没有在多中心的研究中验证^[95-97]^。在所有联合手术治疗的方式中，联合治疗的并发症的发生率不超过70%，专门的医疗中心的死亡率不超过7%（证据水平：强/低等水平证据）。

目前，研究人员对三种治疗方式联合应用是否获益提出质疑^[51]^，最近，一项瑞士研究验证了61例高危患者诱导化疗后行胸膜肺切除术和减量放疗的效果。45例接受EPP的患者的生存期为23个月，而整组患者的生存期为19.8个月^[51]^（证据水平：强/低等水平证据）。总之，目前缺乏最佳综合治疗的数据。

建议

建议将适合联合治疗的患纳入专门的研究中心的前瞻性随机试验中。

### MPM的对症治疗

间皮瘤有较多的临床症状，通过对53例接受顺铂和吉西他滨化疗的间皮瘤患者研究发现，根据EORTC的生活质量问卷调查的评分显示：MPM患者疲倦、呼吸困难、疼痛、失眠、咳嗽和食欲减退等症状比肺癌患者更严重^[98]^。这一部分将讨论间皮瘤患者的常见症状。一项回顾性随机研究评估了间皮瘤的常见症状，结果见补充数据表 5。

**疼痛处理**

如何评估MPM患者的疼痛？

间皮瘤的疼痛由有害因子、神经因子和炎症因子共同作用所致^[99]^。

应用面部表情评分法进行疼痛评分可提高对疼痛的管理（1C级）。

如果患者因疼痛或疾病进展出现认知障碍，可以采用行为评分法，如Doloplus法，来评估疼痛（1C级）。

MPM疼痛的基本处理原则是什么？

建议：间皮瘤的疼痛处理应按照癌症疼痛处理原则（1C级）。

然而，由于因间皮瘤的疼痛机制复杂，除采用阿片类止痛药外，长需使用其它镇痛药。顽固性疼痛在常规治疗无效时，可采用专家疼痛控制或专家姑息疗法建议（1C级）。

必要时，根据专家的建议，在权衡利弊的情况下，采用神经破坏疗法进行止痛（2C级）。

因肿瘤压迫引起的疼痛，可采用姑息性放疗（2C级）。

**呼吸困难的处理**

反复的胸腔穿刺合适吗？

建议：如果在疾病的早期实施了胸膜固定术，或者胸腔积液局限或/和肺萎限不能复张，反复的胸腔穿刺应该避免（1C级）。

对于有复发性胸腔积液且非常虚弱的患者，行反复抽吸或胸腔引流是最为有效的处理方法。（2C级）。

胸膜固定术的作用是什么？

建议：胸膜固定术对预防复发性胸腔积液有效。无菌滑石粉优于其它制剂。（1A级）

应该在什么时候进行胸膜固定术？

建议：通常在疾病早期实施胸膜固定术的疗效最好（1C级），但是在未获得足够的组织用于诊断之前，不能实施胸膜固定术（1A级）。

呼吸困难的治疗方法还有哪些？

建议：小剂量口服吗啡在缓解症状的同时可以减轻焦虑。（1A级）。

吸氧或许有帮助，但是必须在出现血氧饱和度降低时才能使用（1C级）。

减轻呼吸困难的方法还有哪些？

制作一个简易的风扇，在脸上吹出凉风，通过三叉神经减轻对呼吸困难的感知。自助气短管理技术是患者对气短自我控制，在肺癌中证明有效，在MPM患者中尚未得到验证^[100]^。

**其它症状的处理**

这是用于减轻常见的症状的一个简单措施（专家建议）。有关姑息医学的更多信息应向专家咨询。

声明

对于咳嗽症状，应使用镇咳药如可待因糖浆或吗啡。排除或治疗胸部感染、心衰等并发症尤为重要。

厌食、体重减轻和疲劳是许多恶性肿瘤恶病质常见的征象。饮食应注意补充高能量，少食多餐，治疗口腔念珠菌感染，避免脱水和便秘。

通过增减衣服、使用风扇、服用药物如西咪替丁可改善出汗。

吞咽困难或许是由于口腔念珠菌感染或肿瘤对食管的外在压迫所致。氟康唑治疗口腔念珠菌有效。食管支架对肿瘤引起的外在压迫亦有效。

腹水是肿瘤通过膈肌向腹膜腔扩散的征兆，腹腔穿刺可以减轻大量腹水引起的不适，但是需要反复进行。

便秘是由于不活动、进食差造成，也是服用阿片制剂的必然结果。应积极规律地使用通便药。这一征象提示肿瘤通过膈侵入腹膜腔。

呕吐是化疗副作用，止吐药治疗有效。呕吐也可能是阿片类镇痛药的副作用，更换药物可能有效。

**MPM患者的心理治疗**

间皮瘤患者常常表现出易怒、抑郁、淡漠等症状。专门的间皮瘤电话咨询报告显示，患者及家属需要关于疾病、治疗方案、获益和医学法学问题的准确信息。

建议

专科护士、心理或精神服务、石棉互助小组可提供帮助（1C级）。

## STATEMENT OF INTEREST

Statements of interest for A. Scherpereel and P. van Houtte can be found at www.erj.ersjournals.com/misc/statements.dtl

## ACKNOWLEDGEMENTS

The affiliations for the Task Force members are as follows. A. Scherpereel: Dept of Pulmonary and Thoracic Oncology, Hospital of the University (CHRU) of Lille II, Lille, France. P. Astoul: Dept of Pulmonary and Thoracic Oncology, Hoˆ pital Sainte-Marguerite, Universite´ de la Me´diterrane´e, Marseille, France. P. Baas: Dept of Thoracic Oncology, The Netherlands Cancer Institute, Amsterdam, The Netherlands. T. Berghmans: Intensive Care and Dept of Thoracic Oncology, Institut JulesBordet, Brussels, Belgium. H. Clayson: Hospice of St. Mary of Furness, Ulverston, Cumbria. P. de Vuyst: Pulmonary Dept, Hoˆ pital Erasme, Brussels, Belgium. H. Dienemann: Thoraxklinik am Universita¨tsklinikum, Heidelberg, Germany. F. GalateauSalle: Dept of Pathology, CHRU de Caen, Caen, France. C. Hennequin: Dept of Cance´rologie-radiothe´rapie, Hoˆpital Saint-Louis, Paris, France. G. Hillerdal: Lung Medicine, Karolinska Hospital,

Stockholm, Sweden. C. Le Pe´choux: Dept of Radiotherapy, Institut Gustave Roussy, Villejuif, France. L. Mutti: Oncologia Clinica, Vercelli, Italy. J-C. Pairon: INSERM, Unit 955, Cre´teil, France. R. Stahel: Policlinic of Oncology, University Hospital of Zurich, Zurich, Switzerland. P. van Houtte: Dept de R adio-oncologie, Institut Jules-Bordet, Brussels, Belgium. J. van Meerbeeck: Dept of Respiratory Medicine and Thoracic Oncology, University Hospital, Gent, Belgium. D. Waller: Dept of Thoracic Surger y, Glenfield Hospital, Leicester, UK. W. Weder: University Hospital, Division of Thoracic Surgery, Zurich, Switzerland.

This article has supplementary data accessible from www.erj.ersjournals.com

本文补充数据可从www.erj.ersjournals.com获取

Note: Reprinted with permission from the copyright holder ©ERS Journals Ltd 2010

注：本文附表 1-5得到版权所有者©ERS Journals Ltd 2010复制许可

**附表 1:** 美国胸科医生学会的推荐级别（Guyatt et al, Chest 2006; 129(1): 174-81） Grading Recommendations from the American College of Chest Physicians (ACCP) (Guyatt et al, Chest 2006; 129(1): 174-81)

分级建议/描述	获益vs风险及负担	证据的方法学质量	意义
1A/强烈推荐，高水平的证据	获益明显大于风险和负担，反之亦然	严谨的随机对照研究或确切的观察研究	强烈推荐，在大多数情况下可以完全应用到患者
1B/强烈推荐，中等水平的证据	获益明显大于风险和负担，反之亦然	较严谨的随机对照研究	强烈推荐，在大多数情况下可以完全应用到患者
1C/强烈推荐，低水平的证据	获益明显大于风险和负担，反之亦然	观察性研究或者病例报告	强烈推荐，但是当更高级别的证据出现时或许会修改
2A/中等推荐，高水平的证据	获益和风险相当	随机对照研究无重大的缺陷或单方面的观察研究	弱的推荐，根据患者的情况不同和社会价值观不同采用不同的方式
2B/中等推荐，中等水平的证据	获益和风险相当	随机对照研究（不一致的结论或方法学有缺陷)	较弱推荐, 根据患者的情况不同和社会价值观不同采用不同的方式
2C/中等推荐，低水平/很低水平的证据	无法评估获益风险、负担，或许比较的接近	观察性研究或者病例报告	很弱的推荐，其它的可选的治疗方式或许也同样合理

**附表 2:** 恶性胸膜间皮瘤的分期^[28]^ Summary of TNM classification in malignant pleural mesothelioma ^[28]^

T1	同侧胸膜
T1a	无脏层胸膜侵犯
T1b	侵及脏层胸膜
T2	同侧肺、膈肌，脏层胸膜融合
T3	纵隔胸膜、脂肪、胸壁，心包壁层
T4	对侧胸膜、穿透膈肌累及腹膜，助骨，广泛胸壁侵犯，纵隔侵犯，心脏，臂丛，椎骨，心脏，恶性胸腔积液
N0	无局部淋巴结转移
N1	同侧支气管/肺门淋巴结转移
N2	同侧肺门和/或隆突下淋巴结转移
N3	对侧纵隔、肺门、同侧/对侧斜角肌/锁骨上淋巴结转移
M0	无远处转移
M1	远处转移

**3:** 评估化疗在恶性胸膜间皮瘤中疗效的Ⅱ期及Ⅲ期研究 Phase Ⅱ and Ⅲ studies assessing the efficacy of chemotherapy in malignant pleural mesothelioma

方案	评估患者数	病理(上皮样/其它)	复发率(%)	中位生存期(月)	生活质量
Ⅲ期随机试验					
顺铂/培美曲塞(5)	226	154/72	41	12, 1	无
顺铂	222	152/70	17	9, 3	
顺铂/雷替曲塞(6)	126	94/32	24	11, 4	有
顺铂	124	75/49	14	8, 8	
积极对症处理(31)	136	99/37	无比较	7, 6	有
ASC联合MVP ou VNR	273	198/75		8, 5	
Ⅱ期随机试验					
顺铂/丝裂霉素C (32)	35	24/11	26	7, 7	无
顺铂/多柔比星	35	24/11	14	8, 8	
多柔比星/环磷酰胺/咪唑羧酰胺(33)	36	16/20	11	7, 0	无
多柔比星/环磷酰胺	40	21/19	13	5, 8	
多柔比星(11)	15	9/23	0	-	无
环磷酰胺	16		0		
非随机研究					
顺铂为基础的化疗：单药化疗					
顺铂(34)	14	-	36	-	无
顺铂(35)	21	-	14	12, 0	无
顺铂(36)	35	-	14	7, 5	无
顺铂(37)	24	13/10	13	5	无
L-NDDP (脂质体) (38)	33	23/10	(42% biopsy -)	11, 2	无
顺铂为基础的化疗:联合化疗					
顺铂/VP16 (39)	25	-	24	-	无
顺铂/VP16 (40)	26	13/14	12	-	无
顺铂/VBL (41)	20	13/7	25	-	无
顺铂/丝裂霉素C/VBL (42)	39	-	21	6, 0	有症状
顺铂/5氟尿嘧啶/甲酰四氢叶酸/丝裂霉素C/依托泊苷(43)	45	33/12	38	16, 0	有症状
顺铂/丝裂霉素C/干扰素*α*2a (44)	43	24/19	23	11, 5	无
顺铂/丝裂霉素C/干扰素*α*2b (45)	19	19/0	11	15, 0	无
顺铂/丝裂霉素C/干扰素*α* (46)	23	16/4	0	12, 0	有症状
顺铂/干扰素*α*2b/他莫昔芬(47)	39	25/11	19	8, 7	No
顺铂/干扰素*α*(48)	30	17/13	27	15, 0	无
顺铂/干扰素*α*2a (49)	12	11/2	42	16, 5	有症状
顺铂/干扰素*α*2a (50)	26	14/12	40	12, 0	无
顺铂/5氮胞啶(51)	36	22/14	14	6, 4	无
顺铂/吉西他滨(52)	21	13/8	48	9, 5	有症状
顺铂/吉西他滨(53)	30	26/6	13.3	9, 6	有
顺铂/吉西他滨(54)	53	42/11	32	11, 2	有
顺铂/吉西他滨(55)	35	22/13	26	13, 0	无
顺铂/吉西他滨(56)	26	26/0	23	19.5	有症状
顺铂/吉西他滨(57)	50	25/25	12	10, 0	无
顺铂/吉西他滨/长春瑞滨(58)	12	7/5	58	11	无
顺铂/依立替康(59)	15	10/5	40.0	6, 5	无
顺铂/依立替康/丝裂霉素C (12)	43	32/11	37	10.8	有
顺铂/多柔比星/丝裂霉素C(60)	23	18/6	22	10, 5	有症状
顺铂/多柔比星/丝裂霉素C/博来霉素/透明质酸酶(61)	27	22/5	44	15, 0	有症状
顺铂/多柔比星/环磷酰胺(62)	23	14/9	30	13, 9	无
顺铂/多柔比星(63)	19	-	42	12, 0	无
顺铂/多柔比星(64)	24	5/21	25	10, 0	有症状
顺铂/多柔比星/干扰素*α*2b (65)	35	19/16	29	9, 3	无
顺铂/表柔比星(66)	63	43/20	19	13, 3	无
顺铂/吉西他滨随后为米托蒽醌/甲氨蝶呤/丝裂霉素C(67)	54	44/10	30	13	无
顺铂/长春瑞滨(68)	54	40/14	30	16, 8	无
卡铂为基础的化疗					
卡铂(69)	17	-	12	-	有症状
卡铂(70)	9	-	22	-	无
卡铂(71)	31	-	16	8, 0	无
卡铂(72)	40	-	7	7, 1	无
卡铂/吉西他滨(73)	50	34/17	26	15, 2	有症状
卡铂/吉西他滨(74)	21	20/1	33	25.5	有症状
卡铂/吉西他滨/多柔比星脂质体(75)	173	116/45	32	13	无
卡铂/干扰素*α*2a (76)	15	11/4	7	6, 1	无
卡铂/异环磷酰胺/依托泊苷/WBHT (77)	27	16/9	19	17, 7	无
卡铂/培美曲塞(78)	102	80/22	19	12.7	无
卡铂/培美曲塞(79)	76	57/19	25	14	无
奥沙利铂为基础的化疗					
奥沙利铂/雷替曲塞(80)	70	46/24	20	7, 4	有症状
奥沙利铂/雷替曲塞(81)	11	10/1	45	-	无
奥沙利铂/吉西他滨(82)	25	16/9	40	13, 0	无
奥沙利铂/长春瑞滨(83)	26	13/12	23	8, 8	有
蒽环类为基础的化疗(无顺铂)					
多柔比星/干扰素*α*2a (84)	25	-	16	11, 0	无
多柔比星/异环磷酰胺(85)	24	15/9	32	7, 0	无
多柔比星/异环磷酰胺(86)	17	-	13	7, 9	无
脂质体多柔比星(脂质体阿霉素)(87)	31	17/14	6	13, 0	无
脂质体多柔比星(阿霉素)(88)	24	14/10	0	8, 5	无
脂质体多柔比星(阿霉素)(89)	15	-	7	-	无
地托比星(90)	35/21	31/4	42.9 (在21例可评估的患者中)	23, 0	无
表柔比星(91)	51	17/35	15	9, 2	无
表柔比星/异环磷酰胺(92)	17	-	6	6, 0	无
表柔比星/白介素-2 (93)	21	12/9	5	10, 0	无
表柔比星(94)	21	-	5	7, 5	无
表柔比星/吉西他滨(95)	26	-	14	13, 3	无
表柔比星/吉西他滨大剂量(96)	23	15/8	13	9, 3	有
小剂量	45	31/14	7	5, 7	
柔红霉素脂质体(97)	14	9/5	0	6, 1	无
吡柔比星(98)	35	21/14	9	10, 5	无
米托蒽醌(99)	29	-	7	-	无
米托蒽醌(100)	40	25/16	3	4, 5	无
美诺立尔(101)	22	-	5	-	无
抗代谢物为基础的化疗					
米托蒽醌/甲氨蝶呤/丝裂霉素C(102)	22	14/3	32	13, 5	有症状
甲氨蝶呤/干扰素*α*/干扰素*γ*1b (103)	26	17/9	27	17, 0	无
甲氨蝶呤(104)	62	42/20	37	11, 0	无
依达曲沙(105) 依达曲沙/甲酰四氢叶酸	20 38	13/7 23/15	25 16	9, 6 6, 6	无
曲美沙特(106)	51	38/13	12	5, 0-8, 9	无
培美曲塞(107) sans AF/B12 avec AF/B12	43 21	45/19	10 16	8, 0 13, 0	有
培美曲塞/吉西他滨(108)	108	69/39	20	8/10	无
Pralatrexate (109)	16	11/5	0	7	无
5氮胞啶(110)	41	30/11	17	6, 7	无
5氮胞啶(111)	15	-	0	4, 7	无
5氟尿嘧啶(9)	20	7/13	5	5, 0	无
雷替曲塞(112)	24	21/3	21	7, 0	无
卡培他滨(113)	26	15/11	4	4, 9	无
吉西他滨(114)	17	9/8	0	4, 7	无
吉西他滨(115)	27	18/9	7	8, 0	无
CB 3717 (116)	18	-	6	-	无
烷化剂为基础的化疗					
异环磷酰胺(117)	26	18/8	4	10, 0	无
异环磷酰胺(118) A组(2, 3 g/m^2^) B组(1, 2 g/m^2^)	13 16	28/1	38 6	8, 0 9, 0	无
异环磷酰胺(119)	39	10/29	3	6, 9	无
异环磷酰胺(120)	26	20/6	8	6, 5	无
异环磷酰胺(121)	38/21	21/17	6	7, 0	无
异环磷酰胺/干扰素*α*2a(122)	39	-	21	10, 0	无
环磷酰胺(123)	13	3/10	23	6, 0	有症状
替莫唑胺(124)	27	17/10	4	8, 2	有
安沙可林(125)	20	-	5	6, 2	无
长春新碱为基础的化疗					
长春瑞滨(126)	29	17/12	24	10, 6	有
去乙酰长春酰胺(127)	21	14/7	0	-	无
去乙酰长春酰胺(128)	20	-	6	-	无
长春碱(129)	20	-	0	3, 0	无
长春新碱(130)	23	19/4	0	7, 0	无
长春氟宁(131)	65	55/10	14	10.8	无
拓扑异构酶抑制剂为基础的化疗					
依托泊苷(静脉注射) (132) 依托泊苷(oral)	47 41	8/39 18/23	4 7	6, 7 8, 5	无
依托泊苷(133)	22	11/8	5	17, 0	无
托泊替康(134)	22	15/7	0	7, 6	无
依立替康(135)	28	17/11	0	9, 3	无
紫杉醇为基础的化疗					
多西他赛/依立替康(136)	15	8/7	0	8, 5	无
紫杉醇(137)	33	23/8	9	5, 0	无
紫杉醇(138)	23	14/10	0	9, 0	无
多西他赛(139)	31	-	10	12, 2	无
多西他赛(140)	19	-	5	4, 0	无
免疫调节剂为基础的化疗					
白介素-2 IP (141)	22	19/3	55	18, 0	无
白介素-2 IP+SC (142)	31	22/9	23	15, 0	无
白介素-2 IV+SC (143)	29	20/9	7	12, 0	无
r干扰素*α*2b IM (144)	13	-	8	15, 5	无
干扰素*α*2a SC (145)	25	-	12	-	无
干扰素*γ* IP(146)	89	71/18	19	-	无
干扰素*β* IM (147)	15	-	0	9, 1	无
干扰素*γ*+巨噬细胞IP (148)	17	16/3	12	29, 2	无
牝牛分枝杆菌(149)	16	10/6	38	10, 5	无
镇静剂(19)	40	36/4	0	7, 7	无
吉非替尼(21)	43	-	4	6, 8	无
甲磺酸伊马替尼(22)	25	20/5	0	13, 3	无
厄洛替尼(25)	63	28/35	0	10	无
其它					
丝裂霉素C (150)	19	11/8	21	-	无
地吖醌(151)	20	-	0	5, 9	无
Acivicine (152)	23	5/18	0	7, 0	无
豹蛙酶(153)	81	50/55	5	6, 0	无
二线化疗					
多柔比星(9)	11	3/8	9	4, 5	无
ZD0473 (8)	43	-	0	6, 7	有
奧沙利鉑/雷替曲塞(10)	14	8/6	0	3, 2	无
多柔比星(11)	6	-	0	-	无
环磷酰胺	5				
培美曲塞(13) 培美曲塞/卡铂	28 11	22/6 11/0	21 18	9.8 8.6	无
培美曲塞(18)联合 最佳支持疗法	123 120	90/33 86/34	18.7% 1.7%	8.4 9.7	有
顺铂/依立替康/丝裂霉素C(12)	10	8/2	20	7.3	有
厄洛替尼/贝伐珠单抗(154)	24	16/8	0	5.8	无
Belinostat (155)	13	7/6	0	5	无
长春瑞滨(156)	63	39/24	16	9.6	无

**4:** EORTC应用PET评估治疗效果指南 EORTC guidelines for therapeutic response assessment with PET (29)

完全缓解	肿瘤体积完全缩小，与周围正常组织无法鉴别
部分缓解	一个周期的化疗后SUV值至少减少15%-25%，或者多于一个周期化疗后SUV值减少大于25%。
稳定	SUV值增加不到25%或减少不到15%并且没有明显的肿瘤摄取（＜20%的长径）
进展	SUV值增加25%或者明显摄取增加(20%的长径）)或者出现新的转移灶。

**5:** 恶性胸膜间皮瘤的常见症状(Nowak AK et al, J Clin Oncol 2004;22(15):3172-80) Most common symptoms in malignant pleural mesothelioma (Nowak AK et al, J Clin Oncol 2004;22(15):3172-80)

症状	有症状的患者（n=80)	百分比
呼吸困难	77	96%
疼痛	73	91%
咳嗽	33	41%
体重减轻	33	41%
食欲减低	20	25%
多汗	14	18%
恶心	11	14%
疲乏	10	13%
吞咽困难	9	11%
便秘	6	8%
腹水	6	8%
呕吐	4	5%
转移性疼痛	4	5%
